# AMYAND’S HERNIA: OUR EPERIENCE AND REVIEW OF LITERATURE

**DOI:** 10.1590/0102-6720201700040014

**Published:** 2017

**Authors:** Gunjan DESAI, SUHANI, Prasad PANDE, Shaji THOMAS

**Affiliations:** 1 Department of Gastrointestinal Surgery, Lilavati Hospital and Research Center, Bandra Reclamation, Bandra West, Mumbai, Maharashtra;; 2 Department of General Surgeryb, All India Institute of Medical Sciences, New Delhi;; 3 Department of Surgeryd, Lady Hardinge Medical College and Smt. Sucheta Kriplani Hospital, New Delhi, India.

**Keywords:** Appendicitis, Hernia, Surgical mesh., Apendicite, Hérnia, Telas cirúrgicas.

## INTRODUCTION

When a normal, inflamed or perforated appendix is found as a content in an incarcerated inguinal hernia, it is called the Amyand’s hernia^1^. The incidence varies from 0.19-1.7% of reported hernia cases^2^. Appendicitis in Amyand’s hernia is believed to be caused by extra luminal compression and can mimic appendicitis or complicated inguinal hernia. Since both inguinal hernia and acute appendicitis are clinical diagnosis, a preoperative radiological diagnosis is usually not available^3^. The clinical importance lies in the fact that it can result in various complications due to delayed diagnosis and mortality has been reported in range of 6-15%^3^ ^,^ ^4^. The most important determinant of treatment is the presence or absence of appendicitis and periappendiceal abscess^5^. Use of mesh is traditionally contraindicated in cases of an inflamed or perforated appendix. However, case series have been published with mesh repair, mainly due to the availability of potent antibiotics and biological meshes^6^ ^,^ ^7^. We here present our experience of three diverse cases of Amyand’s hernia and review its present literature in brief.

## CASES REPORT

**FIGURE 1:**
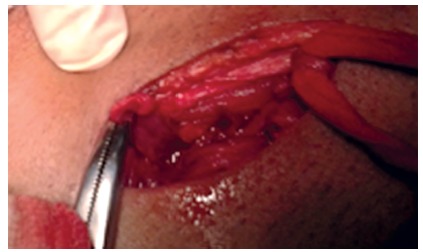
Inflammed appendix in Amyand hernia

**CASE 1 -** A 58 year old male presented to the surgical emergency with complaint of swelling in right groin for one month, with pain in swelling and irreducibility for two days. There were no features of intestinal obstruction. A strangulated right inguinal hernia was present. The patient was taken up for right inguinal exploration/hernia surgery after resuscitation. On opening the hernial sac, inflamed appendix was present as the content of the hernia without any perforation or pus in the sac (Figure 1). An appendicectomy was done followed by herniotomy and herniorrhaphy using the modified Bassini repair. Patient had an uneventful recovery and was asymptomatic at one year follow up.

**CASE 2 -** A 42 year old male patient presented to the surgical emergency with complaint of swelling in right groin for six months with pain in swelling and irreducibility since last five days. On examination, tachycardia and an irreducible right inguinal hernia with localized tenderness and skin redness was present. With a preoperative diagnosis of strangulated right inguinal hernia, the patient underwent right inguinal exploration. On opening the sac, purulent fluid was present and a perforated appendix was present in the sac. Appendicectomy with lavage was done followed by herniotomy and herniorapphy using modified Bassini’s repair. Suction drain with two limbs was placed in right groin area with one limb beneath the fascia transversalis and other limb above the sheath closure. Postoperatively, patient had an uneventful recovery except a superficial surgical site infection which was managed with dressing. 

**FIGURE 2:**
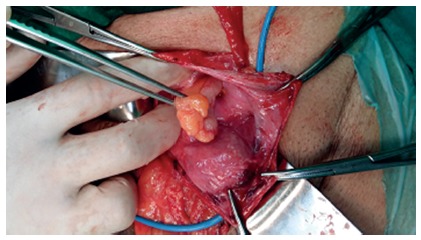
Amyand hernia with normal appendix

**CASE 3 -** A 68 year old male presented to the surgical outpatient department with complaint of swelling in right groin for two months with self limiting episodes of pain and irreducubililty, without any obstructive features. On examination, a partly irreducible right inguinal hernia was present .The patient was subsequently taken up for right open inguinal hernia surgery (Lichtenstein repair). Intraoperatively, on opening the hernial sac a normal appendix was present as the content of the hernia (Figure 2) without any perforation or pus in the sac. An appendicectomy was not done and it was simply pushed in the peritoneal cavity followed by sac closure and polypropylene mesh placement to cover the defect. The patient was asymptomatic at six month follow up. 

## DISCUSSION

When a normal or inflamed or perforated appendix is incarcerated in an inguinal hernia, it is called the Amyand’s hernia named after Claudius Amyand, who, on December 6, 1735, performed appendectomy for the treatment of an 11-year-old boy who presented with a right non-reducible inguinal hernia with the content as appendix. This was the first reported case of appendicectomy, performed via groin approach^1^ ^,^ ^8^.

The incidence is 0.19-1.7% of reported hernia cases^2^. The incidence of appendicitis within an inguinal hernia is even rarer; with an estimated rate at 0.07-0.13%^6^. It is three times more common in children, due to the patency of the processus vaginalis. It is more common in males and when in females is seen in postmenopausa^3^. It is seen mostly in right side. Very few cases of left-sided Amyand’s hernia been described in the literature associated with situs inversus or mobile cecum^7^. 

Different theories have been proposed for the occurrence of Amyand’s hernia. Due to a long appendix pointing towards the groin or loose peritoneal reflections and redundant cecum, the appendix may reach the hernia and get stuck in the sac^9^ ^,^ ^10^.

Appendicitis in Amyand’s hernia is believed to be caused by either extraluminal compression causing edema of appendix with narrowing of the ring along with contraction of abdominal wall muscles causing incarceration and strangulation. The classical intraluminal obstruction of the appendix does not seem to have an important role. However, diffuse peritonitis is considered to be less likely in case of complicated appendicitis because of localization of the contents within the sac^3^ ^,^ ^9^.

The classification of Amyand hernias was first proposed by Losanoff and Basson, and was modified by Rikki, as shown in Table 1 ^11^.

**TABLE 1:** Rikki’s classification of Amyand’s hernia

Classification	Description
Type 1	Appendix within an inguinal hernia - not inflammed
Type 2	Acute appendicitis within an inguinal hernia, no pus or perforation, no abdominal sepsis
Type 3	Acute appendicitis within an inguinal hernia with local or peritoneal pus or sepsis
Type 4	Acute appendicitis within an inguinal hernia with some related or unrelated abdominal pathology
Type - 5 a	Normal appendix within an incisional hernia
Type - 5 b	Acute appendicitis within an incisional hernia, no pus or perforation
Type - 5 c	Acute appendicitis within an incisional hernia, abdominal wall, or peritoneal sepsis or in relation to previous surgery

The most common symptom is a painful groin swelling. Other symptoms can mimic appendicitis or incarcerated, strangulated or obstructed inguinal hernia. On examination, a tender irreducible groin hernia is present. A preoperative radiological assessment is not usually done because of the incarcerated or strangulated presentation which prompts urgent surgery**.** Also as both appendicitis and inguinal hernia are clinical diagnosis, detailed radiological imaging is not warranted^5^ ^,^ ^9^.

Amyand’s hernia can result in various complications such as perforated appendix with periappendicular or intra-abdominal abscess, necrotizing fascitis of the anterior abdominal wall, epididymo-orchitis or testicular abscess, and rarely an in situ arterial thrombosis. Mortality has been reported in range of 6-15%^3^ ^,^ ^4^.

The most important determinant of treatment in Amyand’s hernia is the presence or absence of appendicitis and periappendiceal abscess^5^. Most surgeons accept the notion of preserving the appendix if normal and that this helps in an uneventful postoperative recovery^12^. Johari et al. suggested performing an appendectomy in cases of left sided Amyand’s hernia in all cases, since this appendicitis has an atypical presentation^13^. The management of Amyand’s hernia as advised by Rikki et al. includes mesh repair only in type 1 and type 5a and appendicectomy in all cases of Amyand’s hernia. The remaining types are treated with primary tissue repairs as per Rikki et al. In our case series, one patient had normal appendix and it was just repositioned back in the peritoneal cavity without removing it.

Use of mesh is traditionally contraindicated in cases of an inflamed or perforated appendix and primary tissue repair is advocated. However, case series have been published with mesh repair even in patients with acute appendicitis with no increase in infection rates^6^ ^,^ ^7^. Biosynthetic meshes are the newest meshes which could have a role in these repairs but is not easily available in India as of now^3^. Our approach is to use the mesh if the planes are not contaminated. 

Lower midline laparotomy is advocated in suspected perforation, pelvic abscess or gangrenous acute appendicitis. Vermillion et al. reported the first laparoscopic appendectomy in a case of Amyand’s hernia with appendicitis. As in other abdominal surgeries, the laparoscopic surgery will probably rise as the years progress^14^. 
